# Addressing the recent outbreak of African horse sickness in Lagos, Nigeria

**DOI:** 10.3389/fvets.2023.1160856

**Published:** 2023-03-31

**Authors:** Ridwan Olamilekan Adesola, Adetolase Azizat Bakre, Bashar Haruna Gulumbe

**Affiliations:** ^1^Department of Veterinary Medicine, Faculty of Veterinary Medicine, University of Ibadan, Ibadan, Nigeria; ^2^Department of Microbiology, Faculty of Science, Federal University Birnin-Kebbi, Kalgo, Kebbi, Nigeria

**Keywords:** African horse sickness, Nigeria, Lagos, outbreak, recent

At a time when the world is battling outbreaks of viral diseases such as Ebola, coronavirus disease, and monkeypox, among others, an outbreak of another viral disease, African horse sickness (AHS), also called equine illness, has been reported in Lagos, Nigeria. AHS is caused by a double-stranded RNA virus belonging to the *Reoviridae* family and genus *Orbivirus*. They have (9) nine different immunological subtypes isolated in Nigeria identified by virus neutralization test (1). Nonetheless, there have been some reported interactions between numbers 1 and 2, 3 and 7, 5 and 8, and 6 and 9 serotypes. There have been no reported interactions with other known orbiviruses (2). The virus is resistant to temperature (relatively heat stable), pH (destroyed at pH ≥ 12 or pH < 6), and disinfectant (lipid solvents such as ether). Arthropod vectors carry the disease, particularly *Culicoides species* (biting midges) (3, 4). The contagious, fatal, viscerotropic AHS virus can produce an illness marked by fever, vascular leakage, and a high fatality rate (5). ASH differentials are Equine encephalosis, Trypanosomosis, Piroplasmosis, *Purpura haemorrhagica*, Anthrax, Equine infectious anemia, Equine viral arteritis, and Hendra virus. It affects the equids' blood, tissue fluids, serous exudates, and different internal organs leading to illness in horses (6). AHS might take between 2 and 14 days to incubate, and after 5–7 days following infection, clinical symptoms usually begin to manifest. Subsequently, increased vascular permeability brings about harm to the respiratory and circulatory systems. The illness comes in four different forms: the acute or mixed type, the peracute or pulmonary form, the subacute edematous or cardiac form, and horse sickness fever (7). According to Center for Infectious Disease Research & Policy (CIDRAP) (2004), the *Equidae* family is susceptible to acute or subacute febrile and seasonal viral infection (3). While horses are completely vulnerable to illness, mules and donkeys may be resistant in some cases. In severe outbreaks, horses' mortality rate may surpass 90% rate (5). ASH virus can be studied from unclotted whole blood obtained from affected animals. Further studies like serology, virus isolation, detection, or typing can be carried out on the blood (2).

Africa south of the Sahara has an enzootic spread of the illness rate (5). AHS was reported to be rising in South Africa's surveillance zone as of February 2004. Similarly, the outbreak of the disease has been reported in Asia. For example, recently, an outbreak of the disease has been reported in Thailand (8). In all suspected and confirmed outbreaks, prompt reporting of the discovery, existence, or suspicion of AHS is crucial.

In Nigeria, the last outbreak of AHS was recorded in 2006 in Lagos after its first occurrence in 1971 (9). AHS is one of the serious infectious diseases included on list C of Nigeria's National Livestock Disease Reporting System (9). On 20th December 2022, an AHS outbreak was reported in a horse stable in Ikoyi, Lagos, Nigeria. The outbreak was confirmed at the National Veterinary Research Institute (NVRI), Vom, Nigeria, by RT-PCR diagnostic technique. The characterization of the virus to detect its serotype is still ongoing because of limited molecular characterizing tools in Nigeria. The veterinarian in charge noticed the typical clinical signs of the diseases such as labored breathing, congested ocular mucous membrane, in appetence, yellowish nasal discharges, dullness and fever. Out of 10 horses available on the horse stable, three female horses were affected. The age of the affected mare was 8 years. Supportive therapies like Vitamin C, combination of amino acids, vitamins, and minerals solutions, Procaine penicillin, dihydrostreptomycin, oral electrolyte with glucose, and eye drop, have been administered to other animals as no drug is available to treat the disease. After the confirmation by the NVRI, a prompt alert was sent to the Federal Ministry of Agriculture and Rural Development (FMARD), Nigeria, in order to provide the necessary action to contain the diseases. The Assistant Director of FMARD had addressed all the directors of veterinary service to look out for this outbreak, and any cases should be reported immediately.

Apart from the supportive therapies for the remaining seven horses, no drugs were available to treat the remaining animal. Biosecurity measures and isolation of any horse that shows the clinical signs of AHS should be considered. It was established in the record of the animals that they were vaccinated against AHS with freeze-dried polyvalent live attenuated African horse sickness vaccine at different time interval because the vaccine was imported, but the cause of the outbreak is not known yet. Further investigation should be carried out on this effect. Characterization of the virus strain should be done to ascertain if we are dealing with the same present strains. Although, vaccine failure may occur as was previously in Kano, Nigeria (10). Also, the high endemic of the AHS vector might lead to its incidence in Southwestern Nigeria. Therefore, the ring vaccination method should be implored to prevent the disease's spread to other horse stables and neighboring states. The primary AHS vaccine available in Nigeria is Onderstepoort Biological Product, if all the susceptible animals in Lagos (apart from the horse where the outbreak occurred) and other neighboring states can be vaccinated, it will prepare their immune system and reduce the spread of AHS to them. One of the causes of the outbreak in Africa is our porous borders, the transport of horses or any AHS-susceptible animals should be tested negative before it will be allowed in any state in Nigeria as well. All means of broadcasting should be utilized to inform the public about the outbreak, and they should report any case noticed to the nearby veterinarian. Free ASH testing should be made available to horse owners and susceptible animal owners to check that animals are not incubating the virus. Lastly, Arthropods such as Culicoides should be controlled with insecticides and prevented from a horse stable using a net.

As the outbreak is ongoing, we implore the public, veterinarians, and the Federal Ministry of Agriculture and Rural Development to follow the recommendation provided in this write-up closely to prevent tremendous economic loss, extinction of some species of horses and another pandemic like COVID-19 in animals.

## Author contributions

RA conceived the idea and participated in the writing of the manuscript draft. AB provided supporting data and material for the writing of the manuscript. BG participated in the writing and review. All authors have read and approved the final draft for submission.
